# Cloning, Expression and Purification of *L. Donovani* Specific Antigen for Serodiagnosis of Visceral Leishmaniasis

**DOI:** 10.4172/2155-9929.1000141

**Published:** 2013-01-31

**Authors:** Dinesh Kumar, Puja Tiwary, Anuradha Dube, Jaya Chakravarty, Madhukar Rai, Shyam Sundar

**Affiliations:** 1 Infectious Disease Research Laboratory, Department of Medicine, Institute of Medical Sciences, Banaras Hindu University, Varanasi, U.P, India; 2 Division of Parasitology, Central Drug Research Institute, Lucknow, U.P, India

**Keywords:** ELISA, rBHUP1, Visceral leishmaniasis, rk39

## Abstract

**Background:**

Rapid diagnostic test using rk39 antigen is widely used for visceral leishmaniasis. However it detects anti-rk39 antibodies in 20-32% of endemic healthy individuals. In search for a better biomarker of infection, we identified a protein of molecular weight 70 kDa (BHUP1), specifically recognized by sera of visceral leishmaniasis (VL) patients.

**Methods:**

The protein was cloned as His-tagged fusion protein and purified. We evaluated the sensitivity and specificity of this protein in an enzyme linked immunosorbant assay (ELISA) format in comparison to the rk39 antigen using sera collected from various groups of individuals.

**Results:**

The sensitivity of rBHUP1 was 96.5% compared to 98.8% with rk39. For healthy controls from non endemic and endemic regions, the specificity of rBHUP1 was 100% and 95.6% compared to 100% and 84.9% for rk39, respectively. For other infectious diseases such as malaria, tuberculosis, viral fever, etc., specificity of rBHUP1 was as low as 74.5% when compared to 94% of rk39. At six month and one year follow-up, 74% and 22.5% patients tested positive with rBHUP1, respectively, compared to 97% and 77.4% with rk39 antigen.

**Conclusion:**

Though the high sensitivity and specificity of rBHUP1 antigen for VL and healthy controls would have made it a good diagnostic biomarkers, however, its non-specific reaction with other infectious diseases limit its utility.

## Introduction

Visceral leishmaniasis (VL) is a vector borne disease that causes significant morbidity and mortality. In India, VL is caused by protozoan parasite *Leishmania donovani*, which is transmitted by phlebotomine sand fly. VL is characterized by enlargement of spleen and liver, hypergammaglobulinemia, pancytopenia, fever and weight loss [1,2]. Gold standard for the diagnosis of VL is the demonstration of parasites in splenic or bone marrow smears. VL is the most severe form of leishmaniasis and is fatal, if untreated. According to the WHO, approximately 350 million people are at risk of leishmaniasis and 2 million new cases occur every year [3-5]. Proper control of the disease requires early diagnosis and treatment. Patients suffering from VL have significant increase in both parasite-specific and non specific immunoglobulin belonging to the IgG, IgM and IgA subclasses due to polyclonal activation of the B-cells, and thus antileishmanial antibodies are present in abundance in the sera of these patients. Rapid immunochromatographic test for detection of anti K39 antigen (conserved in the kinesine region of *L. infantum*) is now the most frequently used tool for the diagnosis of VL. Unfortunately 15-32% of healthy individuals living in the endemic region were also tested positive with rk39 based rapid tests [6,7].

Thus, the aim of this work was to identify a parasite antigen which has better specificity and similar sensitivity in the diagnosis of VL. We had earlier reported identification of an antigen (BHUP1) from the lysate of *Leishmania donovani* using proteomics [8]. Now we report the immunoreactivity of the recombinant form of this antigen using ELISA and immunoblotting, and its utility and limitations in the diagnosis of VL by testing human sera from various disease backgrounds.

## Materials and Methods

### Study population

This study was conducted at Infectious Disease Research Laboratory of the Department of Medicine, Institute of Medical Sciences, Banaras Hindu University, Varanasi, and at its Kala-Azar Medical Research Centre (KAMRC) in Muzaffarpur, Bihar, our field site and. The study was approved by the Ethical Committee of the Institute of Medical Sciences, Banaras Hindu University, and Varanasi. Written informed consent was obtained from all participating subjects.

Sera from 202 parasitological confirmed patients with VL, and 100 and 31 after six months and 1-3 years after treatment, respectively, were collected. Controls, with no past history of VL, constituted 114 and 50 healthy individuals living in the endemic and non-endemic regions, respectively, and 129 patients suffering from other febrile illnesses including amoebic liver abscess (n=23), viral fever (n=27), tuberculosis (n=43) and malaria (n=36). The sera were stored at -20°C until use.

### Parasite culture and DNA isolation

A WHO reference strain of *L. donovani* (LEM 138:MHOM/IN/00/DEVI) was grown in medium 199 (Gibco, Grand Island, NY) supplemented with 10% heat inactivated FCS (Gibco), 100 units/ml of penicillin G sodium, and 100 μg/ml of streptomycin sulphate at 25°C in a BOD incubator as described elsewhere [8,9]. *L. donovani* cultures were pelleted and washed with PBS followed by DNA isolation using DNeasy Blood and Tissue Kit (Qiagen, USA) according to the manufacturer’s instructions. DNA was quantitated using a NanoDrop 2000c (Thermo Scientific Inc., Wilmington, DE).

### PCR and insert preparation

An immunogenic fraction was found by screening of *L. donovani* promastigote crude soluble antigens with the different group of serum samples. Protein sequence obtained from MALDI TOF analysis revealed that peptide from the immunogenic protein fraction corresponds to a heat shock protein of molecular weight 70 kDa containing 653 amino acids [8]. Forward primer 5’**GGATCC**ATGACATTCGACGGCGCCATCGGCATCGAC3’ and reverse primer 5’**GAATTC**GTCGACCTCCTCGACCTTGGGGCCAGAGG3’ specific to the hsp70 gene were designed (based on the *L. donovani* hsp70 gene sequence) using OLIGO software (National Bioscience, Plymouth, Minn.) and Gene Runner software (Hastings Software, Inc., Hastings, NY) with the addition of *Bam*H1 and *Eco*R1 restriction sites flanking the forward and reverse primers, respectively.

For PCR amplification, 100 ng genomic DNA of *L.donovani* was used as a template with 1.5 U Taq DNA polymerase (New England Bio labs, UK), 10X Taq DNA polymerase buffer, 1.5 mM MgCl_2_, 25 μM dNTPs and 10 pM of both the primers. The PCR program included an initial denaturation at 95°C for 5 min, followed by 30 cycles of denaturation at 95°C for 1 min, annealing at 65°C for 1.5 min, extension at 72°C for 2 min and a final extension at 72°C for 10 min. The amplified PCR product was electrophoresed in a 1.5% (w/v) ethidium bromide (Merck, Mumbai) stained agarose gel (Sigma-Aldrich, St Louis, USA). The amplified DNA fragment was excised from the gel and purified using a QIAquick Gel Extraction kit (Qiagen, USA) following the manufacturer’s recommendations.

### Construction of cloning vector pTZ57R/T - hsp70 gene

Eluted fragment of hsp70 gene was ligated in pTZ57R/T (T/A) cloning vector using T4 DNA ligase (InsTAclone™ PCR Cloning Kit, Fermentas, Lithuania). The ligated product was transformed into competent DH5α *E. coli* cells and plated on Luria Broth agar plates (HiMedia, Mumbai) containing 50 μg/ml ampicillin. The transformants were screened for the presence of recombinant plasmid under similar PCR condition as mentioned previously. Isolated positive clones were sequenced (Eurofins laboraroty, Bangalore, India) and submitted (GenBank accession no. JQ990221.1).

For sub cloning, plasmid from positive clones was isolated using QIA prep spin miniprep kit (Qiagen) as per manufacturer’s protocol and digested with *Bam*H1 and *Eco*R1 to release hsp70 gene fragment and ligated at the *Bam*H1 and *Eco*R1 restriction site of bacterial expression vector pET28a (Novagen city and country), using T4 DNA ligase (Fermentas) with 1:2 molar ratio of vector and DNA fragments. The reaction mixture was transformed into competent *E. coli* Rosetta strain by standard heat shock protocol, plated on LB agar plate with 50 μg/ml kanamycin (USB, USA) and incubated at 37°C overnight.

### Expression and purification of recombinant protein

The confirmed clone was picked from the plate and inoculated in 10 ml of Luria Broth (LB) media containing 34 μg/ml of chloramphenicol and 50 μg/mL kanamycin and allowed to grow overnight at 220 rpm. Next day 1% of the inoculum was seeded into 200 ml of LB media supplemented with 34 μg/mL of chloramphenicol and 50 μg /mL kanamycin and grown at 37°C until OD_600_ reached 0.6, after which protein expression was induced with 1 mM IPTG (Sigma-Aldrich, St Louis, USA), and further grown for 4 to 16 hrs at 18°C. The cell pellet of 200 ml induced culture was resuspended into 10 mL of lysis buffer containing 1:200 dilution of protease cocktail inhibitor (Sigma-Aldrich, St Louis, USA) and 1% Triton X-100, incubated for 30 min with 1 mg/mL lysozyme (Sigma-Aldrich, St Louis, USA). The suspension was sonicated for 10 × 20 sec (with 20 s intervals between each pulse) on ice. The sonicated cells were centrifuged at 15,000 g for 30 min, and the supernatant was incubated at 4°C for 1 h with 2 mL of Ni–NTA Superflow resin (Qiagen, Hilden, Germany) previously equilibrated with lysis buffer. The suspension was loaded in chromatography column. After washing with 10 column volumes of wash buffer, the purified recombinant protein was eluted with a linear imidazole concentration gradient starting from 10 mM up to 250 mM. The eluted fractions were analyzed by 12% SDS–PAGE and the gels were stained with coomassie brilliant blue R-250 (Sigma–Aldrich, St. Louis, USA). The purified protein content of the fractions was estimated by the BCA method using BSA as standard.

### Immunoblots using patient and control sera

For western blot, sera from VL individuals were also collected at the end of successful treatment (post treatment). 27 serum samples from each of the study groups (pre treatment, post treatment, six month follow-up, NEHC, EHC), were selected randomly. For each study group, three different sera pooled together to form nine panels.

Purified recombinant protein was subjected on 12% SDS-PAGE and transferred to PVDF (0.45 μm pore size, Millipore, USA) membrane using a Bio-Rad transblot apparatus. The protein was immunoblotted as described previously [10] with few modifications. The membrane was further treated with sera (1:100 in PBS) of different study groups, for 1 hour at 37°C. Alkaline phosphatase conjugated with goat anti human IgG (1:1000) was used as a secondary antibody. At the end, color was developed using BCIP-NBT as a substrate (Promega, USA).

### ELISA with recombinant antigen

Assay was done as described elsewhere with some modifications [11]. ELISA using the purified recombinant antigen was first standardized by using different concentration of antigen following the checkerboard method. The optimum concentration of the antigen was found to be 50 ng/well and in 1:400 serum dilutions. Microtitre plates (Nunc, USA) were coated with recombinant BHUP1 protein (50 ng/well) in carbonate buffer (pH 9.6) for overnight at 4°C and blocked with 1% Bovine serum albumin (BSA) in 1X PBS for 2 hours at 37°C. Plates were incubated with sera (1:400 dilutions) at 25°C for 1 hour, followed by HRP conjugated goat anti human IgG (1:30,000) for an hour, and developed with tri-methylene benzidine (TMB, Promega) as a substrate. The reaction was stopped in 1N H_2_SO_4_, and OD_450_ was measured on an ELISA plate reader (Molecular Devices, Spectromax 190, USA). The absorbance is expressed as mean ± standard deviation (SD) of triplicate samples. The cutoff value was determined as 2 SDs above the mean absorbance of NEHC (n=44). The rk39 antigen was used as a standard comparator and ELISA experiments were run simultaneously with same samples at same dilution of primary and secondary antibody similar to BHUP1 antigen ELISA.

### Statistical analysis

Data were analyzed using licensed Statistical Package for Social Sciences Version 16.0 for Windows (SPSS Inc., Chicago IL, USA). Statistical significance of differences in mean absorbance was determined by student’s *t*-test. *A* p value of <0.05 was considered as significant.

## Results

### Cloning, sequence analysis and purification of hsp70 gene

**Figure 1:**
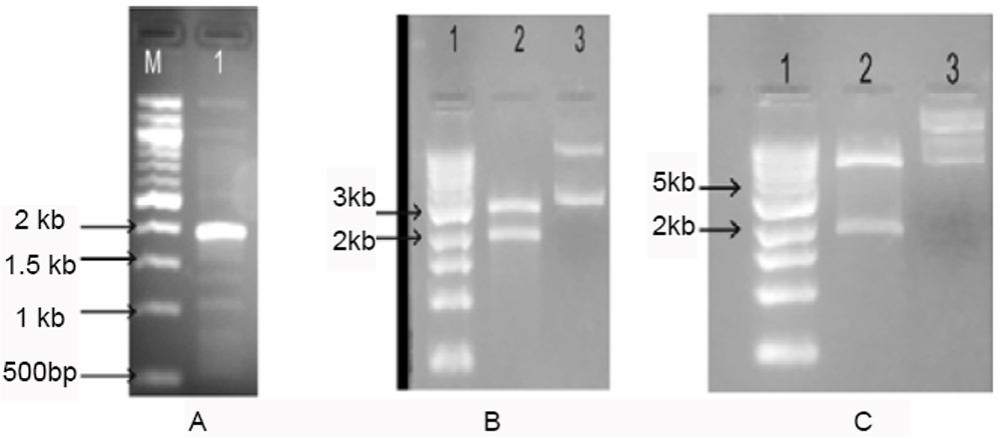
(A) The amplified BHUP1 gene (1959BP) from promastigotes parasite DNA by PCR. Lane 1: 1kb DNA ladder and Lane 2: amplified hsp70 gene. (B) Electrophoretic analysis of pTZ57R/BHUP1 constructs. Lane 1: 1kb DNA ladder, Lane 2: plasmid digested with BamH1/EcoR1 restriction enzymes and Lane 3: uncut plasmid. (C) Ligation of clone BHUP1 gene into expression vector pET28a (+). Lane 1: DNA Ladder, Lane 2: digested pET28a vector and Lane 3: Ligated pET28a vector.

**Figure 2:**
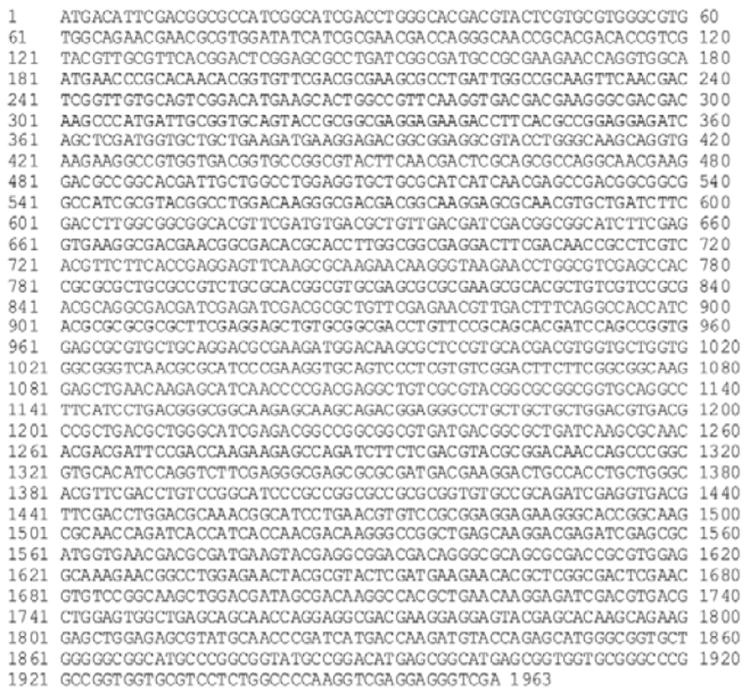
Sequencing result of *L. donovani* BHUP1 clone sequence.

**Figure 3:**
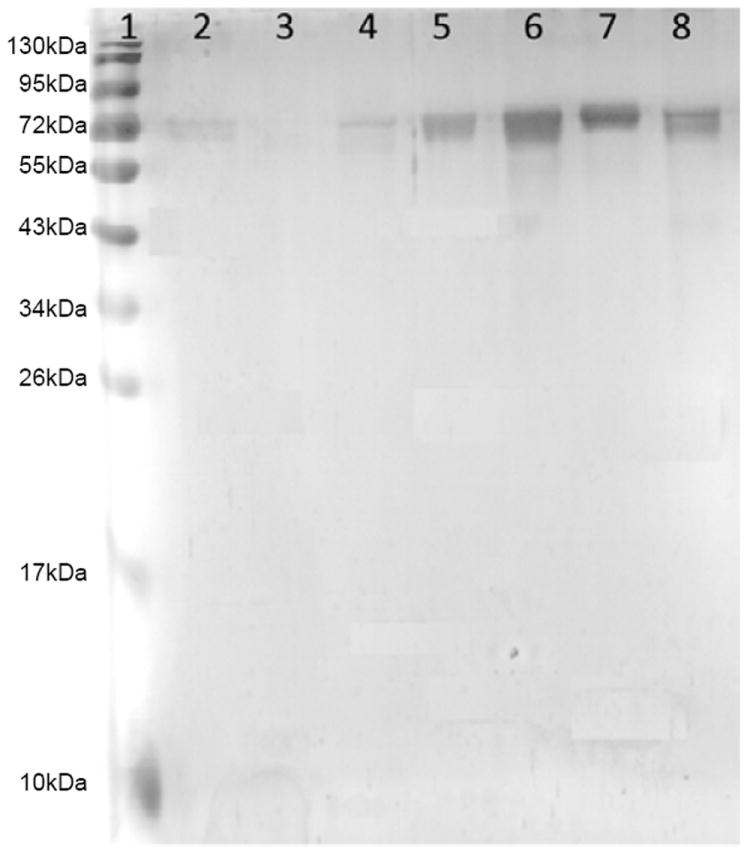
SDS PAGE of purified protein (BHUP1) stained with silver. Lane 1: 10-180kDa Protein marker, Lane 2: Flow through (FT), Lane 3/4: wash fractions, Lane 5/6/7/8: Elution of protein.

Using the primers, a 1959 base pair hsp70 gene fragment flanked by *Bam*HI and *Eco*RI restriction sites was amplified (Figure 1A). The amplified product was gel purified and cloned in the pTZ57R/T vector (Figure 1B). Recombinant clones were selected by colony PCR using same primers. Clones were confirmed by *Bam*HI/*Eco*RI digestion, and by sequence analysis (Figure 2). The amplified hsp70 gene fragment was 99% identical with *L. donovani* HSP70 in GenBank. The hsp70 gene fragment was sub cloned into pET28a+ (Figure 1C) and recombinant protein was purified (Figure 3).

### Immunoblot

**Figure 4:**
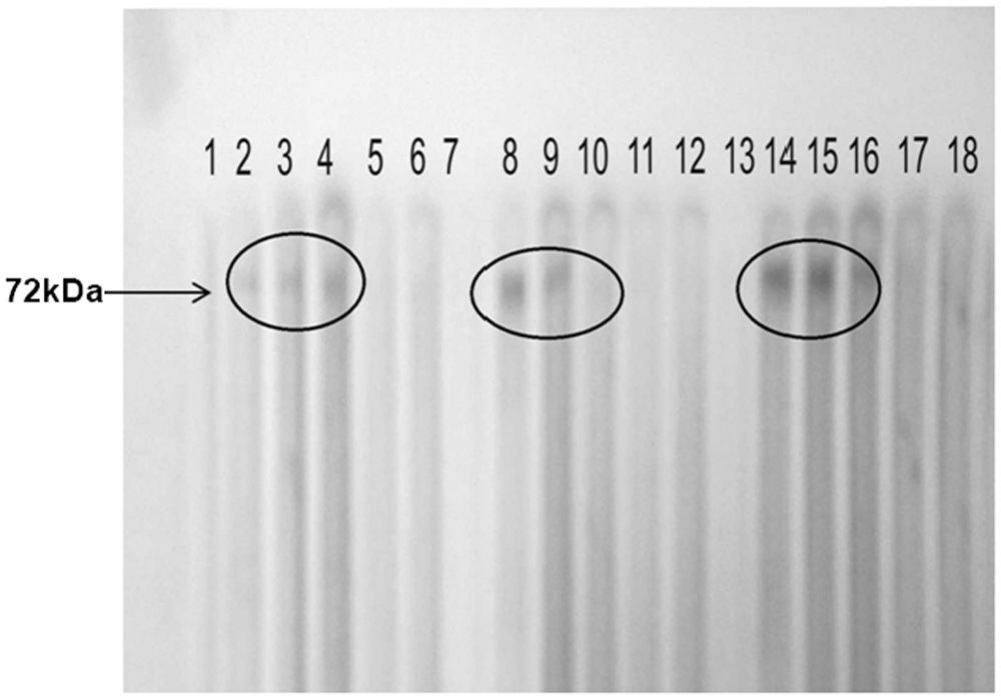
Western blot of BHUP1 protein with a panel of serum. Lane 2/8/14: pool serum of three pretreated patients, Lane 3/9/15: pool serum of three post treated patients, Lane 4/10/16: pool serum of three six month follow up, Lane 5/11/17: pool serum of three endemic control and Lane 6/12/18: pool serum of three non endemic control.

Sera from various subject groups were tested for the presence of antibodies against rBHUP1 using immunoblot. Both the pre and post treatment serum of VL patients recognized this antigenic protein while none of the sera from endemic or non endemic healthy controls recognized it (Figure 4).

### ELISA

Result of ELISA with both rBHUP1 and rk39 shown in table 1. The sensitivity of rBHUP1 protein was 96.5% (CI 93.0, 98.30) with VL sera, while the specificity was 100% (CI 95.8, 100) and 95.6% (CI 90.1, 98.1) in case of non endemic and endemic controls respectively. However, specificity of rBHUP1 protein was low (74.5%) in case of other infectious disease, that included 14 cases of malaria, 11 cases of tuberculosis and 8 cases of viral fever, while 94% specificity was shown with rk39.

## Discussion

For the serodiagnosis of VL various strategies have been used during the past several years and there have been waves of vivacity favoring one technique over others [12]. Previous investigations reported VL specific antibody in the patient’s sera due to activation of B cells [13,14]. This led to search of a serologic biomarker for the diagnosis of VL.

Many rapid diagnostic antigens have been tested for the diagnosis of VL. Several vendors are marketing rk39 antigen based rapid tests which has a lower sensitivity in East Africa, recently rk28 antigen based diagnostic tools (rapid as well as ELISA) have shown to perform better in African countries [15], though the results were similar to rk39 antigen in India [16].

The sensitivity and specificity of rk39, which is widely used for diagnosis of VL worldwide, was reported as 98% and 89% respectively in a meta-analysis [17]. Several other kinesin-related protein antigens such as K26, K9, KRP42, and KE16 have been tested for their serodiagnostic potential [18-20].

Our endeavor was to discover a *L. donovani* specific antigen, which would be highly sensitive and specific. This led to the discovery of BHUP1 antigen as a candidate diagnostic antigen. In the study with antigen drawn from promastigotes, this antigen appeared to be very promising with very high degree of specificity in all classes of controls [9], similar to the ones used in the present study. The next logical step was to get it characterized and make a recombinant antigen and repeat the experiments to confirm the earlier findings. Unfortunately even though the sensitivity was high and comparable to that with rk39, and specificity was excellent with endemic controls, which has always been the problem with rk39 assays, its cross reactivity to the tune of >25% with other febrile illness, tuberculosis in particular, questions its utility. This low specificity indicates the ubiquitous nature of this antigen. It has been reported that short peptides may induce a specific response while large peptide may evoke a non-specific response due to specificity of antigen [21].

This rBHUP1 antigen has comparable sensitivity with gold standards antigen like rk39. In case of endemic healthy controls, rBHUP1 was much more specific than rK39. In patients with long term follow up (1-2 years after cure), rBHUP1 antigen was much more specific and only 22.5% individuals were positive compared to 77.4% with rK39. Thus, rBHUP1 antigen can differentiate between healthy and diseased individual with greater clarity.

In conclusion, BHUP1 antigen has clear superiority over rK39 as far as diagnosis of VL is concerned and turns negative in majority of the patients 1-2 years after cure, however, it has a limitation as there is significant cross-reaction with individual suffering from tuberculosis and other diseases.

## Figures and Tables

**Table 1:** Comparison of sensitivity and specificity of BHUP1 and rk39 antigen ELISA.

Subjects	BHUP1 (95% CI)	rk39 (95% CI)
Non endemic healthy (n=50)	100% (CI 95.8, 100)	100% (CI 95.8, 100)
VL (n=202)	96.05% (CI 93.0, 98.30)	98.8% (CI 95.9, 99.7)
Endemic healthy (n=114)	95.60% negative (CI 90.1, 98.1)	84.5% negative (CI 76.8, 90.5)
Different disease (n=129)	74.4% specific (CI 64.6, 81.6)	94% specific (CI 89.1, 97.7)
Six month follow up (n=100)	74% positive (CI 64.6, 81.6)	97% positive (CI 91.5, 98.9)
>1 Year follow up (n=31)	22.5% positive (CI 11.4, 39.8)	77.4% positive (CI 60.2, 88.6)

## References

[1] Adler S (1964). Leishmania. Adv Parasitol.

[2] Peters W, Killick-Kendrick R (1987). The Leishmaniasis in Biology and Medicine.

[3] Baily GG, Nandy A (1994). Visceral leishmaniasis: more prevalent and more problematic. J Infect.

[4] Herwaldt BL (1999). Leishmaniasis. Lancet.

[5] (1997). Special Programme for Research and Training in Tropical Disease, Leishmaniasis. Thirteenth Programme Report.

[6] Sundar S, Sahu M, Mehta H, Gupta A, Kohli U (2002). Noninvasive management of Indian visceral leishmaniasis: clinical application of diagnosis by K39 antigen strip testing at a kala-azar referral unit. Clin Infect Dis.

[7] Sundar S, Singh RK, Maurya R, Kumar B, Chhabra A (2006). Serological diagnosis of Indian visceral leishmaniasis: direct agglutination test versus rK39 strip test. Trans R Soc Trop Med Hyg.

[8] Kumar S, Kumar D, Chakravarty J, Rai M, Sundar S (2012). Identification and characterization of a novel Leishmania donovani antigen for serodiagnosis of visceral leishmaniasis. Am J Trop Med Hyg.

[9] Maurya R, Mehrotra S, Prajapati VK, Nylen S, Sacks D (2010). Evaluation of blood agar microtiter plates for culturing leishmania parasites to titrate parasite burden in spleen and peripheral blood of patients with visceral leishmaniasis. J Clin Microbiol.

[10] Towbin H, Staehelin T, Gordon J (1979). Electrophoretic transfer of proteins from polyacrylamide gels to nitrocellulose sheets: procedure and some applications. Proc Natl Acad Sci U S A.

[11] Hommel M, Peters W, Ranque J, Quilici M, Lanotte G (1978). The micro-ELISA technique in the serodiagnosis of visceral leishmaniasis. Ann Trop Med Parasitol.

[12] Srivastava P, Dayama A, Mehrotra S, Sundar S (2011). Diagnosis of visceral leishmaniasis. Trans R Soc Trop Med Hyg.

[13] Gidwani K, Picado A, Ostyn B, Singh SP, Kumar R (2011). Persistence of Leishmania donovani antibodies in past visceral leishmaniasis cases in India. Clin Vaccine Immunol.

[14] Londner MV, Feinsod FM, Faris R, Rosen G, el Said S (1988). Persistence of human leishmanial antibodies in an endemic area of visceral leishmaniasis in El Agamy, Egypt. Eur J Epidemiol.

[15] Pattabhi S, Whittle J, Mohamath R, El-Safi S, Moulton GG (2010). Design, development and evaluation of rK28-based point-of-care tests for improving rapid diagnosis of visceral leishmaniasis. PLoS Negl Trop Dis.

[16] Vaish M, Bhatia A, Reed SG, Chakravarty J, Sundar S (2012). Evaluation of rK28 antigen for serodiagnosis of visceral Leishmaniasis in India. Clin Microbiol Infect.

[17] Chappuis F, Rijal S, Soto A, Menten J, Boelaert M (2006). A meta-analysis of the diagnostic performance of the direct agglutination test and rK39 dipstick for visceral leishmaniasis. BMJ.

[18] Mohapatra TM, Singh DP, Sen MR, Bharti K, Sundar S (2010). Compararative evaluation of rK9, rK26 and rK39 antigens in the serodiagnosis of Indian visceral leishmaniasis. J Infect Dev Ctries.

[19] Sivakumar R, Sharma P, Chang KP, Singh S (2006). Cloning, expression, and purification of a novel recombinant antigen from Leishmania donovani. Protein Expr Purif.

[20] Takagi H, Islam MZ, Itoh M, Islam AU, Saifuddin Ekram AR (2007). Short report: production of recombinant kinesin-related protein of Leishmania donovani and its application in the serodiagnosis of visceral leishmaniasis. Am J Trop Med Hyg.

[21] Wallace GR, Ball AE, MacFarlane J, el Safi SH, Miles MA (1992). Mapping of a visceral leishmaniasis-specific immunodominant B-cell epitope of Leishmania donovani Hsp70. Infect Immun.

